# STIM1/SOX2 proteins are co-expressed in the tumor and microenvironmental stromal cells of pancreatic ductal adenocarcinoma and ampullary carcinoma

**DOI:** 10.1186/s12957-024-03356-y

**Published:** 2024-03-26

**Authors:** Dina Sweed, Sara Mohamed Abd Elhamed, Hayam Abdel Samie Aiad, Nermine Ahmed Ehsan, Aiat Shaban Hemida, Marwa Mohammed Dawoud

**Affiliations:** 1 Pathology Department, National Liver Institute, Menoufia University, Shibin Al Koom, Egypt; 2 Pathology Department, Faculty of Medicine, Menoufia University, Shibin Al Koom, 32511 Egypt

**Keywords:** Ampullary carcinoma, BCL2, Immunohistochemistry, Pancreatic ductal adenocarcinoma, SOX2, STIM1

## Abstract

Pancreatic ductal adenocarcinoma (PDAC) and ampullary carcinoma (AAC) are lethal malignancies with modest benefits from surgery. SOX2 and STIM1 have been linked to anticancer activity in several human malignancies. This study included 94 tumor cases: 48 primary PDAC, 25 metastatic PDAC, and 21 primary AAC with corresponding non-tumor tissue. All cases were immunohistochemically stained for STIM1 and SOX2 and results were correlated with clinicopathologic data, patient survival, and BCL2 immunostaining results. Results revealed that STIM1 and SOX2 epithelial/stromal expressions were significantly higher in PDAC and AAC in comparison to the control groups. STIM1 and SOX2 expressions were positively correlated in the primary and metastatic PDAC (P = 0.016 and, P = 0.001, respectively). However, their expressions were not significantly associated with BCL2 expression. SOX2 epithelial/stromal expressions were positively correlated with the large tumor size in the primary AAC group (P = 0.052, P = 0.044, respectively). STIM1 stromal and SOX2 epithelial over-expressions had a bad prognostic impact on the overall survival of AAC (P = 0.002 and P = 0.001, respectively). Therefore, STIM1 and SOX2 co-expression in tumor cells and intra-tumoral stroma could contribute to the development of PDAC and AAC. STIM1/SOX2 expression is linked to a bad prognosis in AAC.

**Supplementary Information:**

The online version contains supplementary material available at 10.1186/s12957-024-03356-y.

## Introduction

Periampullary adenocarcinoma is a malignant tumor that develops from the ampulla of Vater either from the pancreas, duodenum, or the distal end of the common bile duct (CBD) [1]. The most common type of periampullary adenocarcinoma is pancreatic ductal adenocarcinoma (PDAC), which is the seventh largest cause of cancer mortality, whereas ampullary adenocarcinoma (AAC) is the second most prevalent type [2].

Pancreatic ductal adenocarcinoma and other non-pancreatic periampullary carcinomas are characterized by aggressive course and metastatic behavior which were demonstrated in nearly 80% of patients. In addition, pancreaticoduodenectomy (Whipple’s operation), the only potentially curative intervention, is not advisable in patients presented with disseminated or advanced tumor stage [3]. Moreover, half of those who underwent surgery and adjuvant therapy developed liver metastasis [4].

Gemcitabine, the treatment of choice for advanced PDAC, has a limited role in improving the survival rate owing to the unique chemoresistance of pancreatic cancer cells [5, 6]. The mechanism of chemoresistance is multifactorial resulting from interaction among pancreatic tumor cells and tumor microenvironment, tumor stemness, the evolution of apoptosis, and the deregulation of calcium channel pathways [7]. Evasion of apoptosis might be crucial in the progression of PDAC and AAC. Additionally, apoptotic protein dysregulation is a major factor in the emergence of chemoresistance in PDAC [8]. B cell lymphoma-2 (BCL-2) is a crucial anti-apoptotic protein that has been shown to control Ca2 + translocation across the ER membrane via store-operated calcium channels (SOCs) [9].

In hypoxic environments, hypoxia inducible factor-1α (HIF-1α) increases PDAC progression by increasing stromal interaction molecule 1 (STIM1) expression in tumor tissue, potentially affecting the prognosis [10, 11]. STIM1 is a major component of SOCs [12]. STIM1 has been linked to the progression and spread of human malignancies via cell cycle arrest and anti-apoptotic activities [10, 13]. However, there has been limited research on the role of STIM1 in PDAC, and no previous studies have demonstrated STIM1’s relevance in AAC.

Similarly, the expression of sex-determining region Y-box2 (SOX2) promotes prostate and breast cancer progression by inhibiting apoptosis and increasing cell proliferation [14]. SOX2 has been reported to contribute in tumor stemness and modulate epithelial-mesenchymal transition (EMT) in a subset of human pancreatic tumors [15, 16]. In addition, SOX2 could be expressed in ampullary preneoplastic mucosal epithelium and invasive carcinoma [17].Wuebben et al. reported that the oncogenic and tumor suppressor function of SOX2 is dependent on its optimal level [18]. The limited studies on the expression of STIM1 and SOX2 in PDAC and AAC could hinder understanding their possible pathogenic and therapeutic role in primary and metastatic PDAC and AAC. In addition, the expression of STIM1 in primary and metastatic cancer is a matter of controversy in different cancers [19, 20]. No previous studies focused on the different expression of SOX2 in primary and metastatic tumors.

The anticancer role of SOX2 and STIM1 has been reported in different human tumors [21–23]. Some anti-cancer medications have been shown to trigger cancer cell death through the SOCE pathway. The ability of SOCE to effectively slow the progression of various tumors, including breast, and liver cancer, is supported by the fact that STIM1 inhibition may have therapeutic value [24]. Similarly, clinical trials on SOX2 inhibitors give impressive results with possible medical uses in treating tumors that express SOX2 [25, 26].

The study aims to evaluate the protein expression of STIM1 and SOX2 in primary and metastatic PDAC as well as AAC. This could illustrate their potential role in advanced and metastatic patients.

## Material and methods

This retrospective, case-control study used all available methods and adhered to all applicable ethical rules. It was carried out on periampullary carcinoma cases divided into 94 tumor cases: 48 primary PDAC patients, 25 metastatic PDAC cases as well and 21 primary AAC cases. Any patients who had received chemotherapy prior to surgery were excluded. There was also a control group of 35 non-tumorous tissue cases (23 pancreatic tissue and 12 intestine tissue) and 10 cases of normal pancreatic tissue. The study was according to Helinski guidelines and institutional approval.

Primary cases underwent Whipple’s operation at the Hepatopancreatobiliary Department. For metastatic cases, the cases were either known cases of primary PDAC with metastatic deposits at the time of presentation or presenting as metastatic of unknown origin but later shown to be of primary pancreatic occult location using clinical, radiological, and pathological data.

The patients’ relevant data, including their overall survival, were obtained from their medical files. OS statistics were calculated from the time of illness until the patient’s death or last follow-up for at least a year.

All malignant cases had a histological evaluation [27, 28]. The tumor cases were divided into early (I and II) and advanced (III and IV) pathological stages, as well as two tiers of pathological grades: low (GI and GII) and high (GIII). After excluding neutrophils and macrophages, only the proportion of tumor-infiltrating mononuclear cells (TIMC) was measured for the tumor immune response [29].

Using a manual needle set (Breecher Instrument, USA) with two viable tissue cores from the tumor and one core from the matched non-tumor samples, tissue microarray construction (TMA) was carried out.

### Immunohistochemical studies

Rabbit polyclonal anti-STIM1 diluted as 1:200 (Cat. No. bs-8526R) obtained from BIOSS, Woburn, Massachusett, USA, rabbit polyclonal anti-SOX2 diluted as 1:250 (Cat. No. GB11249) obtained from Service bio, Wuhan, China, and a rabbit monoclonal anti-BCL2 antibody ready to use, (Cat. No. ab32124) obtained from Abcam, Cambridge, UK were used. After deparaffinization and rehydration of the tissues, the antigens were retrieved using a low-PH citrate solution and cooled at room temperature. Primary antibodies were applied to the slides and left incubating at 4 °C overnight. The secondary antibody, anti-polyvalent horseradish peroxidase3,3′diaminobenzidine (DAB), was applied using Ultravision’s detecting tools, and the staining was visualized with a counteract Mayer’s hematoxylin. Positive and negative controls were included. Positive control for STIM1 was gastric tissue, SOX2 was esophageal tissue, and for Bcl2 the tumor-infiltrating lymphocytes (TILs) were used as an internal positive control [30].

### Antibody assessment methods

STIM1 stained positively for brownish granular cytoplasmic staining, whereas SOX2 stained positively for brownish nuclear staining [10, 16]. The expression was assessed in both the epithelial and stromal components. Stromal components included fibroblasts, endothelial cells, and infiltrating inflammatory cells. BCL2 exhibited cytoplasmic and/or membrane staining [31].

The three antibodies were assessed using an H-score, calculated by determining the percentage of cells at each intensity. The following method is used to get the final score: [1 (% cells 1+) + 2 (% cells 2+) + 3 (% cells 3+)]. The final score is between 0 and 300 [32]. Furthermore, tumor cases were divided according to the median of the H-score into low and high expression.

### Statistical analysis

Using IBM SPSS, the chi-square test, Fisher’s Exact (FE), or Monte Carlo correction (MC) tests were used to ascertain the relationship between the qualitative variables. The marginal homogeneity and McNemar (McN) tests were used to determine whether there was a significant difference between two qualitatively paired data. The student t-test (t), Mann Whitney (U), and Kruskal Wallis (H) tests were employed, respectively, to compare quantitative variables that were either normally distributed or abnormally distributed, respectively. To investigate the relation between two variables, the Spearman coefficient was utilized. When the two-tailed P-value is 0.05 or below, it is regarded as statistically significant. The OS data for the patients were assessed using Kaplan-Meier plots and the log-rank test. The factor influencing mortality was verified using univariate COX-regression analysis.

## Results

### The clinicopathological data of the studied groups

PDAC cases are often diagnosed at larger tumor sizes which could interfere with complete surgical resection of the tumor compared to AAC (P < 0.001, and P = 0.002, respectively). In addition, the primary PDAC group showed a significant positive perineural invasion compared to primary AAC group (P < 0.001).

There was no significant difference between primary and metastatic PDAC in terms of clinicopathological characteristics.

The clinicopathological data of the studied groups are summarized in Supplementary Table 1.

### The expression of STIM1 and SOX2 in the studied groups

**Fig. 1:**
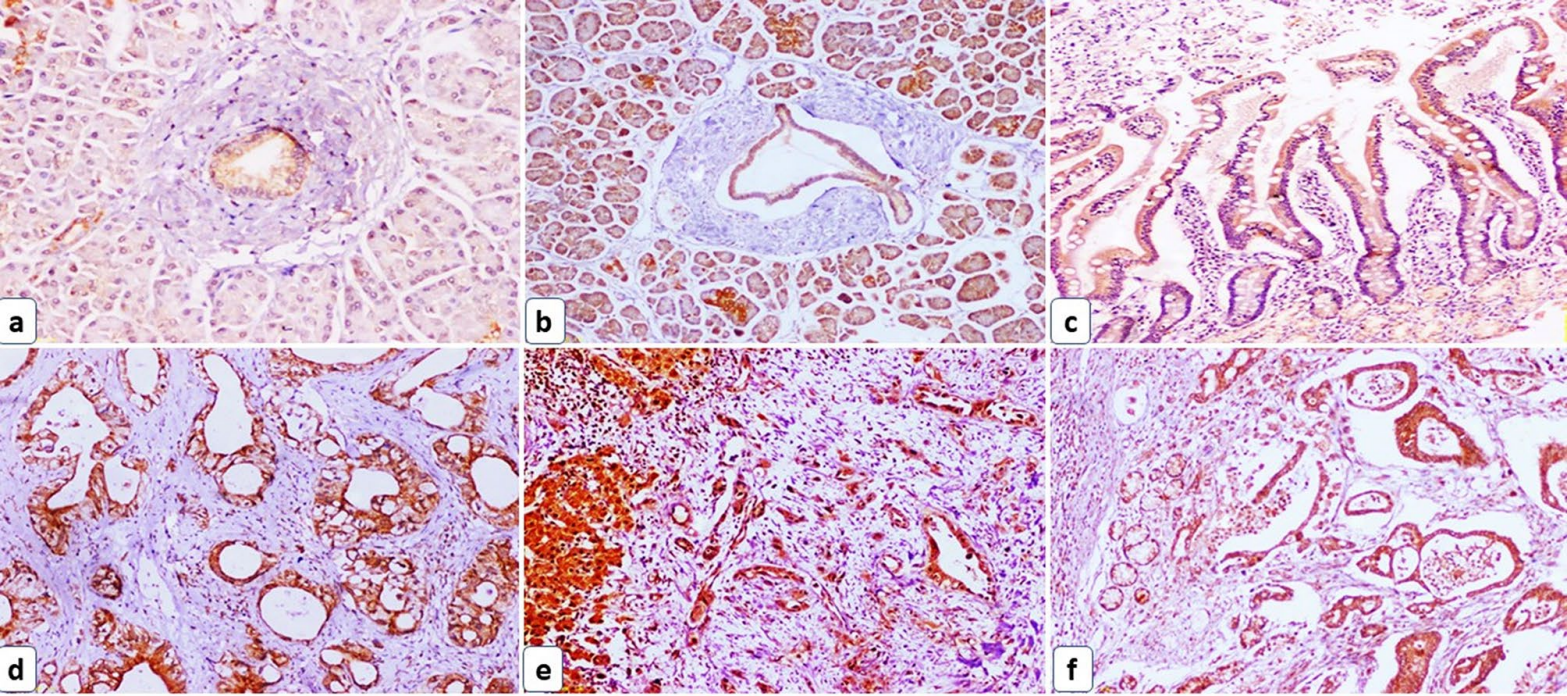
STIM1 immunohistochemical expression in the studied groups. (**a**) A case of normal pancreatic tissue showed weak cytoplasmic STIM1 epithelial expression in the pancreatic duct and focal cytoplasmic STIM1 stromal expression (IHC x200), (**b**) A case of adjacent non-tumor pancreatic tissue showed moderate cytoplasmic STIM1 epithelial expression and focal STIM1 cytoplasmic stromal expression (IHC x100), (**c**) A case of control non-tumor intestinal tissue showed moderate cytoplasmic STIM1 epithelial expression and focal cytoplasmic STIM1 stromal expression (IHC x100), (**d**) A case of primary PDAC showed strong cytoplasmic STIM1 epithelial and stromal expression (IHC x100), (**e**) A case of metastatic PDAC to the liver (on the left side) showed strong cytoplasmic STIM1 epithelial and stromal expression (IHC x100), (**f**) A case of AAC showed strong cytoplasmic STIM1 epithelial and stromal expression (IHC x100)

The epithelial expression of STIM1 was observed in 60% of the normal pancreatic tissue cases, 73.9% of the adjacent non-tumor pancreatic tissue, and in all primary and metastatic PDAC groups. Similarly, 75% of the non-tumor intestinal tissue group showed positive STIM1 epithelial expression while the AAC group showed positive expression, Fig. 1.

**Fig. 2:**
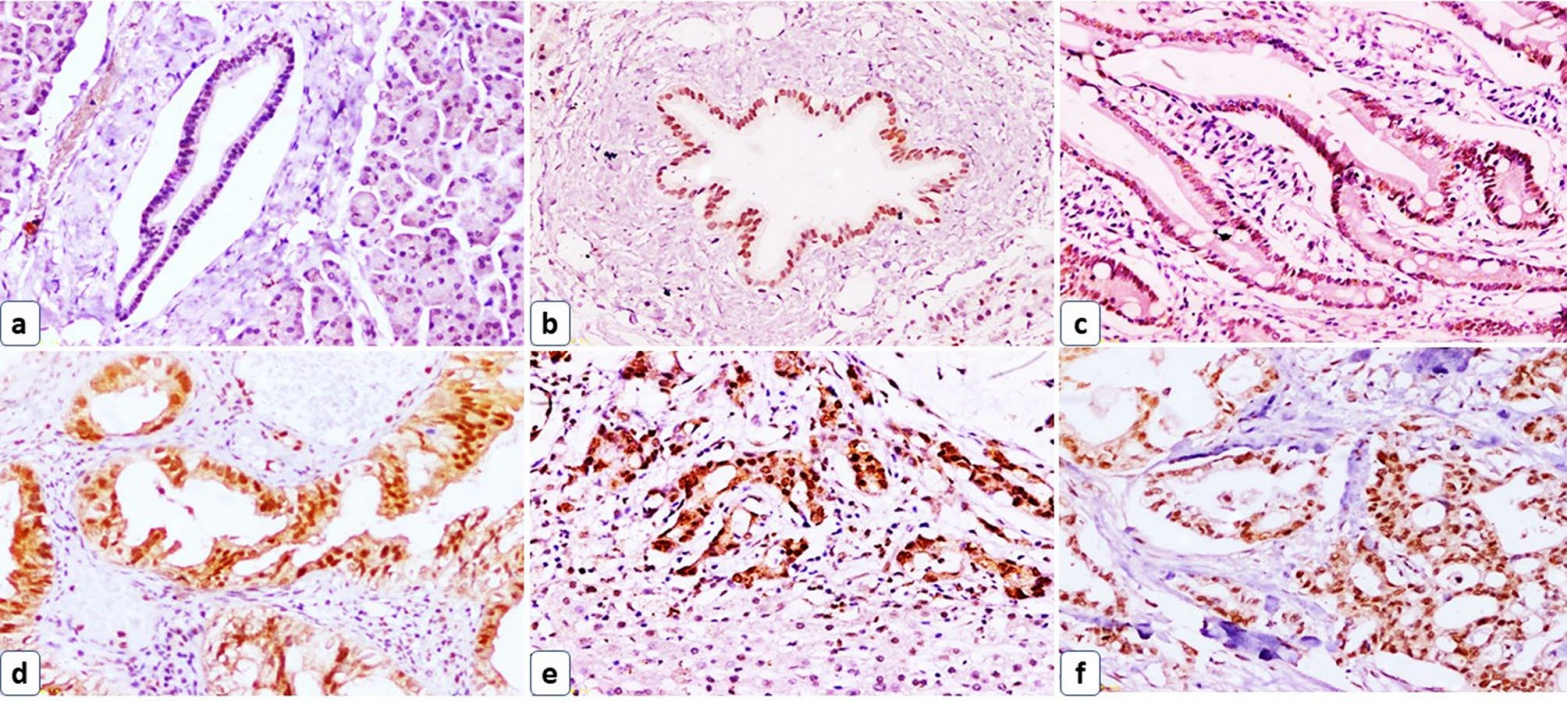
SOX2 immunohistochemical expression in the studied groups. (**a**) A case of normal pancreatic tissue showed mild nuclear SOX2 epithelial expression and negative nuclear SOX2 stromal expression (IHC x200), (**b**) A case of adjacent non-tumor pancreatic tissue showed moderate nuclear SOX2 epithelial expression and negative nuclear SOX2 stromal expression (IHC x200), (**c**) A case of control non-tumor intestinal tissue showed mild nuclear SOX2 epithelial expression and negative nuclear SOX2 stromal expression (IHC x100), (**d**) A case of well-differentiated PDAC showed strong nuclear SOX2 epithelial and stromal expression (IHC x200), (**e**) A case of metastatic PDAC to the liver (down of the plate) showed strong nuclear SOX2 epithelial and stromal expression (IHC x200), (**f**) A case of AAC showed strong nuclear SOX2 epithelial and stromal expression (IHC x100)

Regarding SOX2, 70% of the control pancreatic tissue groups showed SOX2 epithelial expression. 72.9% and 92% of primary and metastatic PDAC groups showed positive expression, respectively. In AAC, 50% of the control non-tumor intestinal tissue group showed positive STIM1 epithelial expression and 61.9% of the AAC group showed positive expression, Fig. 2.

### Comparison of STIM1 and SOX2 expression in different groups

**Fig. 3:**
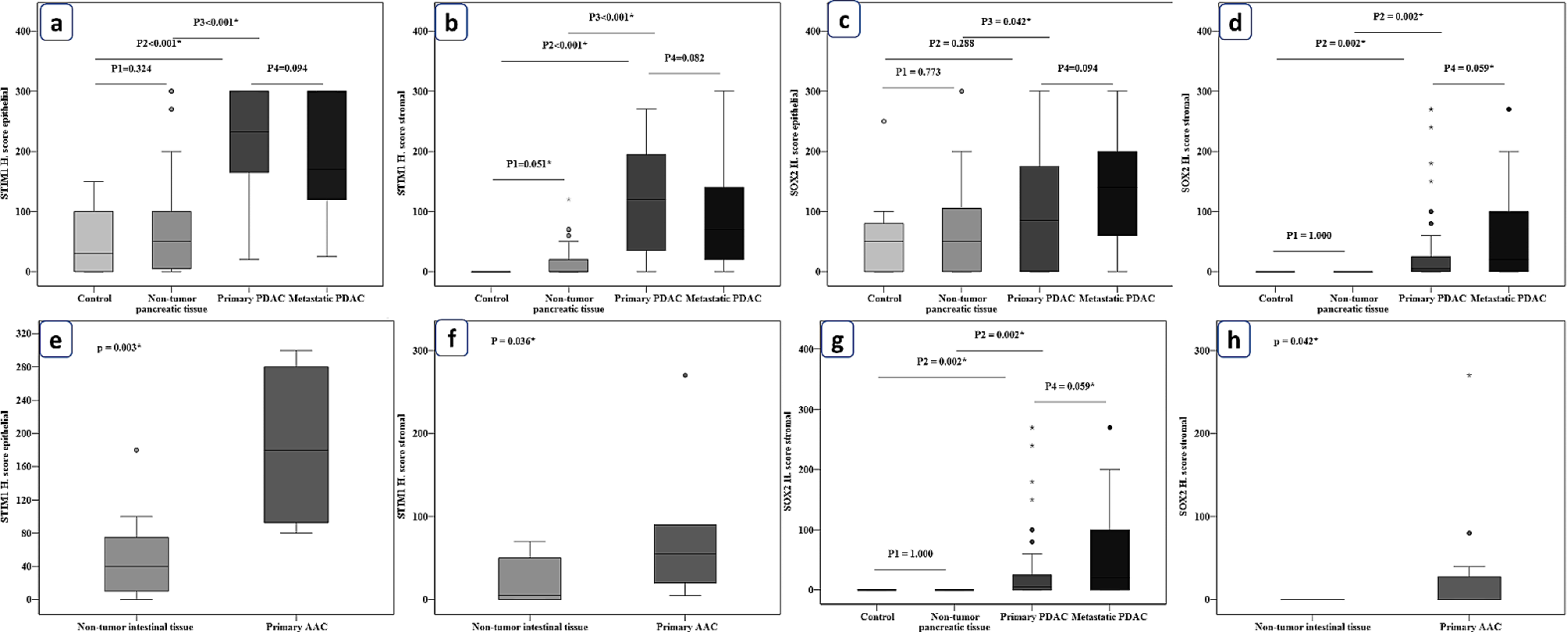
Comparison between STIM1 and SOX2 expression in PDAC and the control groups. (**a**) Comparison between STIM1 epithelial expression in PDAC and the control groups, (**b**) Comparison between STIM1 stromal expression in PDAC and the control groups, (**c**) Comparison between SOX2 epithelial expression in PDAC and the control groups, (**d**) Comparison between SOX2 stromal expression in PDAC and the control groups. (**e**) Comparison between STIM1 epithelial expression in AAC and the control groups. (**f**) Comparison between STIM1 stromal expression in AAC and the control groups. (**g**) Comparison between SOX2 stromal expression in PDAC and the control groups. (**h**) Comparison between SOX2 stromal expression in AAC and the control groups

PDAC had significantly higher STIM1 epithelial and stromal expressions than the groups of control pancreatic tissue (P < 0.001 for both). STIM1 epithelial and stromal expressions did not significantly differ across the primary and metastatic PDAC groups (P = 0.094 and P = 0.082, respectively), Fig. 3a and b.

Primary PDAC had significantly higher SOX2 epithelial expression compared to non-tumor tissue (P = 0.042). The primary and metastatic PDAC groups showed a similar SOX2 epithelial expression (P = 0.094). In addition, primary PDAC had significantly increased SOX2 stromal expression in comparison to the healthy control group and nearby non-tumor pancreatic tissue (P = 0.002 and P = 0.003). When compared to the primary PDAC, metastatic PDAC tended to have more SOX2 stromal expression (P = 0.059), Fig. 3c and d.

In AAC, STIM1 epithelial and stromal expressions were significantly higher compared to the control non-tumor intestinal tissue group (P = 0.003 and P = 0.036), Fig. 3e and f. Primary AAC between and control non-tumor intestinal tissue groups did not significantly vary in terms of SOX2 epithelial expression (P = 0.153). However, stromal expression of SOX2 was significantly increased in AAC than in the non-tumor intestinal tissue group (P = 0.042), Fig. 3.

STIM1 and SOX2 expressions did not statistically differ between the primary PDAC and primary AAC groups.

### The correlation of STIM1 and SOX2 expression in the studied groups

STIM1 and SOX2 epithelial expressions positively correlated in primary PDAC (r = 0.346, P = 0.016). Furthermore, STIM1 and SOX2 stromal expressions positively correlated in metastatic PDAC (r = 0.618, P = 0.001).

### The relationship between STIM1 and SOX2 expression and the anti-apoptotic BCL2 marker

BCL2 was expressed in a range of 4-8.3% of the PDAC and AAC groups. Furthermore, no significant correlations were found between STIM1/SOX2 and BCL2 expression.

### The relationship between STIM1 and SOX2 and the pathological data in PDAC and AAC

**Fig. 4:**
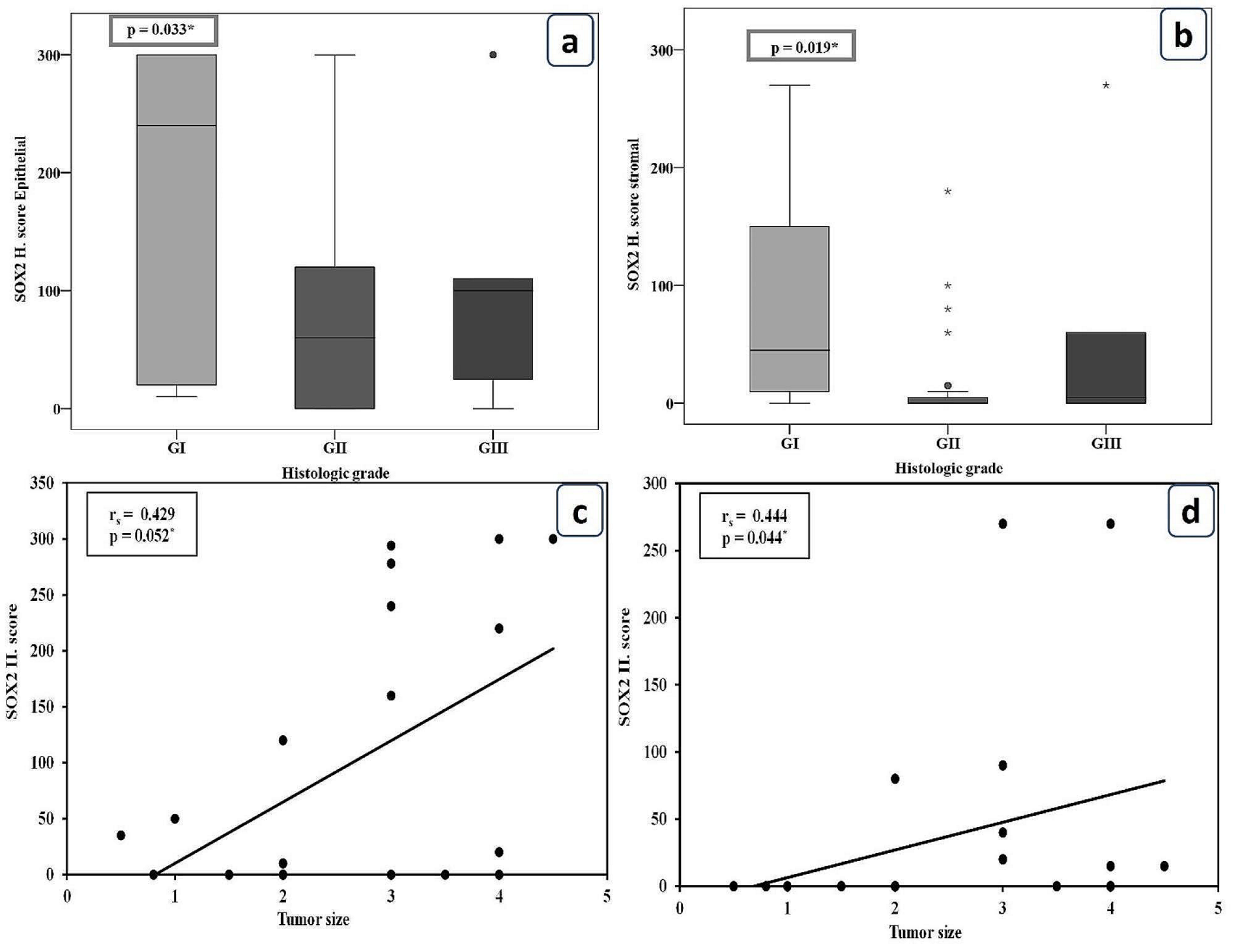
The relationship of SOX2 expressions with the clinicopathological parameters of PDAC and AAC: (**a**) Correlation between SOX2 epithelial expression and tumor grade (P = 0.033) in primary PDAC. (**b**) Correlation between SOX2 stromal expression and tumor grade (P = 0.019) in primary PDAC. (**c**) Correlation between SOX2 epithelial expression and tumor size (P = 0.052) in primary AAC. (**d**) Correlation between SOX2 stromal expression and tumor size (P = 0.044) in primary AAC

Significant associations between SOX2 epithelial and stromal expression and the primary PDAC group’s well-differentiated grade have been found (P = 0.033 and P = 0.019). On the other hand, SOX2 epithelial and stromal expressions were positively correlated with large tumor size in the primary AAC group (P = 0.052 and P = 0.044), Fig. 4.

There was no significant association between STIM1 and SOX2 expression and the clinicopathological parameters of metastatic PDAC.

Tables 1 and 2 showed the detailed relationship between STIM1 and SOX2 and the pathological data in PDAC and AAC.

**Table 1:** Relationship between STIM1 expression and clinicopathological data in the primary PDAC group (No = 48) and AAC (No = 21)

Variables		No	Epithelial STIM1 H-score in PDAC		Test of sig.	No	Epithelial STIM1 H-score in AAC		Test of sig.
					&				&
			Mean ± SD.	Median	p		Mean ± SD.	Median	p
Age (years)	< 56	22	216.59 ± 77.36	200	U = 261.0	12	188.75 ± 89.85	190	U = 46.500
	≥ 56	26	226.54 ± 86.58	255	P = 0.592	9	206.67 ± 71.94	220	P = 0.602
Gender	Male	35	228.0 ± 85.09	260	U = 182.50	14	209.29 ± 83.89	230	U = 35.0
	Female	13	205.77 ± 72.68	200	P = 0.280	7	170.71 ± 74.63	180	P = 0.322
Tumor recurrence	Positive	17	229.12 ± 75.42	215	U = 52.0	6	155.0 ± 60.25	155	U = 5.500
	Negative	7	210.0 ± 91.47	200	P = 0.664	5	240.0 ± 78.74	280	P = 0.082
Tumor size					r= -0.144				r_s_ = -0.032
					P = 0.329				P = 0.890
Histologic grade	GI	10	239.50 ± 59.28	237.5	H = 1.722	3	206.67 ± 30.55	200	U = 24.0
	GII	33	211.52 ± 89.34	200	P = 0.423	17	193.24 ± 89.77	200	P = 0.921
	GIII	5	256.0 ± 60.66	300		1^#^	220	--	
LVI	Present	15	212.67 ± 91.22	200	U = 231.50	5	201.0 ± 101.39	240	U = 36.0
	Absent	33	226.21 ± 78.23	250	P = 0.713	16	195.0 ± 77.72	200	P = 0.780
Pathological stage	Early	29	234.66 ± 72.53	260	U = 227.0	10	185.50 ± 84.93	190	U = 47.50
	Late	19	202.63 ± 92.85	200	P = 0.290	11	206.36 ± 80.41	220	P = 0.605
LNs status	Positive	35	225.29 ± 84.29	250	U = 199.5	10	205.0 ± 80.09	220	U = 47.50
	Negative	13	213.08 ± 77.07	200	P = 0.501	11	187.0 ± 85.64	200	P = 0.605
Perineural invasion	Present	47	225.0 ± 79.90	250	–	10	201.0 ± 79.37	220	U = 52.0
	Absent	1^#^	80		–	11	192.27 ± 86.47	200	P = 0.863

STIM1: Stromal interaction molecule 1, PDAC: Pancreatic ductal adenocarcinoma, AAC: Ampullary adenocarcinoma, LNs: Lymph nodes, T: Tumor, SD: Standard deviation, H-score: Histo-score, U: Mann Whitney test, H: Kruskal Wallis test, r: Spearman coefficient, p: p-value for comparing between the two categories, *: Statistically significant at P ≤ 0.05

#: Excluded from the comparing due to small number of case (n = 1)

**Table 2:** Relationship between SOX2 expression and clinicopathological data in the primary PDAC group (No = 48) and AAC (No = 21)

Variables		No	Epithelial SOX2 H-score in PDAC		Test of sig.	No	Epithelial SOX2 H-score in AAC		Test of sig.
					&				&
			Mean ± SD.	Median	p		Mean ± SD.	Median	p
Age (years)	< 56	22	110.0 ± 116.75	60	U = 283.50	12	108.08 ± 130.47	27.5	U = 47.0
	≥ 56	26	100.77 ± 99.69	95	P = 0.958	9	82.22 ± 114.98	10	P = 0.651
Gender	Male	35	105.86 ± 104.46	90	U = 210.50	14	115.86 ± 133.79	35	U = 43.0
	Female	13	102.69 ± 117.13	40	P = 0.690	7	59.29 ± 90.20	10	P = 0.689
Tumor recurrence	Positive	17	108.24 ± 114.47	90	U = 50.0	6	17.50 ± 21.39	10	U = 12.0
	Negative	7	55.71 ± 41.17	60	P = 0.576	5	120.80 ± 160.91	10	P = 0.662
Tumor size					r_s_= 0.180				r_s_= 0.429
					P = 0.222				P = 0.052^*^
Histologic grade	GI	10	191.00 ± 123.60	240	H = 6.822^*^	3	172.67 ± 150.74	240	U = 20.0
	GII	33	78.64 ± 87.65	60		17	89.35 ± 118.17	20	
	GIII	5	107.00 ± 117.77	100	P = 0.033^*^	1#	0	--	P = 0.616
LVI	Present	15	83.33 ± 95.57	40	U = 206.0	5	115.6 ± 112.77	120	U = 32.50
	Absent	33	114.85 ± 111.46	90	P = 0.351	16	91.19 ± 127.36	10	P = 0.548
Perineural invasion	Present	47	105.53 ± 107.85	90	–	10	146.20 ± 141.9	135	U = 33.50
	Absent	1^#^	80		–	11	52.27 ± 83.05	10	P = 0.132
Pathological stage	Early	29	104.83 ± 113.79	60	U = 275.0	10	65.80 ± 108.62	0.5	U = 35.50
	Late	19	105.26 ± 98.07	100	P = 0.991	11	125.36 ± 131.03	50	P = 0.173
LNs status	Positive	35	95.14 ± 99.64	80	U = 0.199	11	135.82 ± 135.77	120	U = 40.0
	Negative	13	131.54 ± 124.35	120	P = 0.504	10	54.30 ± 92.69	10	P = 0.314

SOX2: Sex-determining region Y-box2, PDAC: Pancreatic ductal adenocarcinoma, AAC: Ampullary adenocarcinoma, LNs: Lymph nodes, T: Tumor, SD: Standard deviation, H-score: Histo-score, U: Mann Whitney test, H: Kruskal Wallis test, r: Spearman coefficient, p: p-value for comparing between the two categories, *: Statistically significant at P ≤ 0.05. #: Excluded from the comparison due to the small number of cases (n = 1)

### The OS study of primary PDAC and AAC groups categorized by clinical, pathological, and IHC marker characteristics

Survival data were available in 52.08% of the primary cases of PDAC and AAC. The mean survival time in the PDAC group was 12.66 ± 8.67 months and the median was 12 months, with 68% dying from the tumor. The mean survival time for AAC was 23.32 ± 13.33 months, with a median of 24 months, 63.6% of cases died from the tumor. In addition, the OS did not differ significantly across the primary PDAC and AAC groups (χ2 = 0.291, P = 0.225). In PDAC, the univariate analysis of OS showed the adverse prognostic impact of tumor recurrence on the patient’s outcome (P = 0.044). However, none of the STIM1 or SOX2 expressions showed a significant impact on the OS of PDAC cases.

**Fig. 5:**
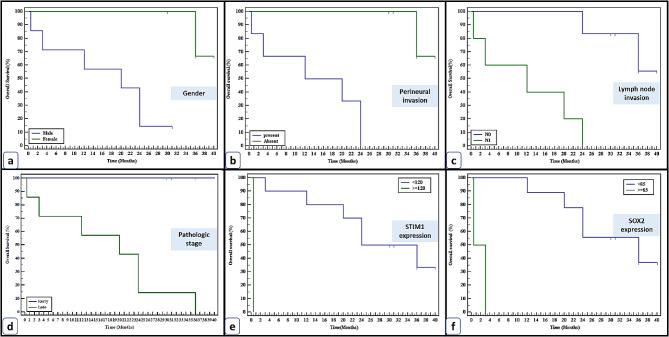
Kaplan-Meier survival curve demonstrating the impact of clinicopathological and STIM1/SOX2 expressions on the overall survival of AAC cases

In AAC, the univariate analysis of OS showed the adverse prognostic impact of the male gender, perineural invasion, positive LNs, and late tumor stage on the patient’s outcome. In addition, STIM1 stromal overexpression and SOX2 epithelial overexpression showed a bad prognostic impact on the patient’s outcome, Fig. 5.

The results of a univariate COX regression analysis for the factors impacting mortality in the primary AAC group revealed that positive LN status is the most predictive parameter influencing mortality in the AAC group (P = 0.015), Table 3.

**Table 3:** Univariate and multivariate COX regression analysis for the parameters affecting morality in the primary AAC group

	Univariate		^#^Multivariate	
	p	HR (95%C.I)	p	HR (95%C.I)
Sex (male)	0.220	63.240(0.08–48065.8)		
Perineural invasion	0.175	116.264(0.12–112,908)		
LNs	0.015^*^	15.396(1.70–139.70)		
Staging (late)	0.171	73.43(0.16–34653.2)		
Stromal STIM1	0.823	0.829(0.16–4.29)		
Epithelial SOX2	0.281	2.583(0.46–14.50)		

HR: Hazard ratio, C.I: Confidence interval, STIM1: Stromal interaction molecule1, SOX2: Sex determining region Y-box2, H-score: Histoscore, C.I: Confidence interval

#: All variables with P < 0.05 was included in the multivariate

*: Statistically significant at P ≤ 0.05

## Discussion

STIM1/SOX2 expression in tumor cells, as well as microenvironment cells, could contribute to PDAC and AAC tumorigenesis.

STIM1 expression increased in PDAC. STIM1 overexpression was seen in about two-thirds of PDAC cases, according to Wang et al. [10]. STIM1 overexpression was shown to be significantly higher in pancreatic tumor cell lines than in normal cell lines [33]. Despite, the limited data regarding the possible oncogenic role of STIM1 in PDAC and AAC, previous studies have postulated the potential oncogenic role of STIM1 in many cancers [21, 34, 35]. The possible oncogenic role of STIM1 is through regulation of SOCE activity, a major modulator of tumor migration and invasiveness, neovascularization, antitumor immunity, inhibition of apoptosis, and induced hypoxia pathways [36–38].

Similarly, SOX2 overexpression was significantly observed in PDAC and AAC cases in comparison with the control groups in the present study. In previous studies, SOX2 was expressed in 20% of PDAC cases while it has a wide range (50- 89.7%) of expressions in AAC [16, 17, 39]. In addition, SOX2 was overexpressed during PDAC progression from in situ to invasive components [15, 18]. In more than 20 different malignancies, SOX2 was found to play an oncogenic function [40]. SOX2 expression was linked to aberrant cross-talks between various signaling pathways that led to the emergence of many malignant characteristics [41].

The lack of any significant difference regarding STIM1 and SOX2 expression between PDAC and AAC could indicate that these tumors may have some similarities in their molecular pathogenesis [42]. Studies have suggested that PDAC and AAC may have different pathogenesis even if both conditions have overlapping symptoms and are being managed in a similar approach. Regarding clinicopathological and molecular aspects, PDAC seems to be the worst [43]. In addition, because of the rarity of AAC in the current and previous studies, there is a lack of related research and comprehension of its characteristics [44].

Furthermore, based on the term “stromal interaction molecule” of STIM1, we proposed a possible function for its expression in the tumor microenvironment cells. STIM1 was shown to be expressed in stromal cells in addition to lung cancer cells [45]. STIM1 may be involved in a wide range of biological functions in non-tumor cells, including immunological cells, endothelial cells, and fibroblasts [46, 47]. We also observed a significant overexpression of SOX2 in the intra-tumoral stromal cells in PDAC and AAC. In some malignancies such as colorectal carcinoma, and lung, stromal SOX2 was found to be linked to a worse prognosis [47–49]. Despite the clinical significance of STIM1 and SOX2 stromal expression is not well elucidated, we could suggest their possible role in the metastatic potential of PDAC and AAC.

Primary and metastatic PDAC showed similar STIM1 and SOX2 expression. Pancreatic cancers metastasize early and thus are molecularly indistinguishable from metastatic tumors [50]. However, the expression STIM1 in metastatic cancer is a matter of controversy. STIM1 was downregulated in metastatic hepatocellular carcinoma (HCC) rather than in primary HCC cells [19]. On the other hand, STIM1 was overexpressed in metastatic gastric cancer compared to the primary sites [20]. Similarly, highly metastatic CRC cell lines have higher STIM1 expression than minimally metastatic cell lines [10]. STIM1 was overexpressed in metastatic melanoma and lung cell lines compared to primary cell line [51, 52]. Regarding SOX2, despite lacking of previous studies, SOX2 modulates several features of tumor metastasis such as EMT, migration, and invasion [53].

PDAC overexpressed epithelial STIM1 had dense desmoplastic stroma which may indicate a crosstalk between the malignant and microenvironmental cells. Desmoplastic stroma facilitates tumor cell growth, invasion, and metastasis, which mediates PDAC progression [54]. STIM1 regulates the activity of transforming growth factor-β (TGF-β) dependent Snail1 transcription genes, which are required for EMT [55]. SOX2 stromal expression in the current study tends to be higher in metastatic PDAC than in the primary ones. SOX2 enhances mesenchymal gene activation while suppressing epithelial genes that enable EMT-induced cancer cell spread [16, 56].

Limited data regarding the correlation of STIM1 and SOX2 in cancer and their possible synergistic impact. STIM1 and SOX2 expressions were found to be positively correlated in the PDAC group. The possible mechanism is through sharing stemness and an antiapoptotic mechanism. Belotte et al. found that STIM1 overexpression influenced the activation of stemness-related genes such as SOX2 [57]. In addition, STIM1 maintained Ca2 + signals in CSCs in a wide range of tumors [58]. STIM1 and SOX2 were discovered to have anti-apoptotic effects in pancreatic cancer cell lines, HCC, and prostate cancer [33, 59, 60]. However, the expression of STIM1 and SOX2 did not show a significant association with the studied anti-apoptotic marker, BCL2 which could be attributed to the limited PDAC and AAC cases expressing BCL2. STIM1 has no significant impact on the apoptosis pathway in gastric cancer [52]. The current debate reflects the variety of linkages between BCL-2 proteins and Ca2 + signaling pathways, as not all targets or mechanisms will be active in all types of cells and situations [61].

There is disagreement regarding the prognostic significance of SOX2 and STIM1 in certain malignancies [16, 18, 57, 62–64]. This was the case in our study. SOX2 expression was linked to the well-differentiated grade of PDAC while associated with poor patients’ survival. In AAC, SOX2 expression was linked to large tumor size and short patient survival. STIM1 stromal expression was linked to short survival in AAC cases. Previous studies found an association of SOX2 with worse prognostic parameters in PDAC [17, 65]. However, Herreros-Villanueva et al. reported a lack of prognostic role of SOX2 in PDAC [16]. This debate may be attributed to SOX2’s ability to regulate the activity of a wide range of genes that can either accelerate or prevent tumor growth [40]. The poor prognostic impact of SOX2 has been found to promote growth, metastasis, and drug resistance in different malignancies [59, 66]. STIM1 may play a site-specific prognostic role in cancer. In pancreatic cancer, STIM1 expression was significantly linked with short survival [10]. Chong et al. reported the absence of a significant relationship between SOX2 expression with the patients’ survival in periampullary cancer [39].

The limitations of this research included lack of advanced microscopes for photographs, and facilities of digital scoring, molecular and invitro experiments and lack of financial resources. Furthermore, there was difficulty obtaining the survival data of the patients.

In conclusion, the expression of STIM1 and SOX2 in PDAC and AAC could indicate a shared pathway; however, not linked to the anti-apoptotic BCL2 expression. Their role in tumorigenesis could be modulated by their direct expression in tumor and non-tumor cells. SOX2 stromal expression could have a role in metastatic PDAC. STIM1 and SOX2 both play a negative prognostic function in AAC. Their prognostic role in PDAC, however, is unclear.

### Electronic supplementary material

Below is the link to the electronic supplementary material.

Supplementary Material 1: **Table 1**: Comparative between PDAC and AAC regarding the clinicopathological data

## Data Availability

The datasets generated and/or analyzed during the current study are not publicly available but are available from the corresponding author on reasonable request.

## Declarations

### Study design

A case-control retrospective study.

## References

[1] Hank T, Klaiber U, Sahora K, Schindl M, Strobel O (2021). [Surgery for periampullary pancreatic cancer]. Chirurg.

[2] Ferchichi M, Jouini R, Koubaa W, Khanchel F, Helal I, Hadad D, Bibani N, Chadli-Debbiche A, BenBrahim E. Ampullary and Pancreatic Adenocarcinoma—a Comparative Study. *J. Gastrointest. Oncol. Vol 10, No 2 (April* 2019*) J. Gastrointest. Oncol*, 2018. 10.21037/jgo.2018.09.09 PMC6465503 31032094

[3] Yeo C (2013). Shackelford’s surgery of the alimentary tract. 7th Ed.

[4] O′Kane GM, Knox JJ (2018). Locally Advanced Pancreatic Cancer: an emerging entity. Curr Probl Cancer.

[5] Malayeri R, Ghassemboland M, Ranjpoor F, Maadi A (2008). Gemcitabine/5-Flourouracil/Leucovorin for the treatment of Advanced Pancreatic Carcinoma. Hematol Oncol Stem Cell Ther.

[6] Melisi D, Budillon A. Pancreatic Cancer: between Bench and Bedside. *Current drug targets*. United Arab Emirates June. 2012;729–30. 10.2174/138945012800564130. 10.2174/138945012800564130 22458518

[7] Zeng S, Pöttler M, Lan B, Grützmann R, Pilarsky C, Yang H (2019). Chemoresistance in Pancreatic Cancer. Int J Mol Sci.

[8] Samm N, Werner K, Rückert F, Saeger HD, Grützmann R, Pilarsky C (2010). The role of apoptosis in the Pathology of Pancreatic Cancer. Cancers (Basel).

[9] Bassett JJ, Robitaille M, Peters AA, Bong AHL, Taing M-W, Wood IA, Sadras F, Roberts-Thomson SJ, Monteith GR (2022). ORAI1 regulates sustained cytosolic free calcium fluctuations during breast Cancer cell apoptosis and apoptotic resistance via a STIM1 independent pathway. FASEB J off Publ Fed Am Soc Exp Biol.

[10] Wang J, Shen J, Zhao K, Hu J, Dong J, Sun J (2019). STIM1 overexpression in Hypoxia Microenvironment contributes to pancreatic carcinoma progression. Cancer Biol Med.

[11] Zoa A, Yang Y, Huang W, Yang J, Wang J, Wang H, Dong M, Tian Y (2022). High expression of Hypoxia-Inducible factor 1-Alpha predicts poor prognosis in pancreatic ductal adenocarcinoma: a Meta-analysis and database validation protocol. Transl Cancer Res.

[12] Roos J, DiGregorio PJ, Yeromin AV, Ohlsen K, Lioudyno M, Zhang S, Safrina O, Kozak JA, Wagner SL, Cahalan MD (2005). STIM1, an essential and conserved component of Store-operated Ca2 + Channel function. J Cell Biol.

[13] Ren R, Li Y (2023). STIM1 in Tumor Cell Death: Angel or Devil?. Cell Death Discov.

[14] Kar S, Niharika; Roy A, Patra SK (2023). Overexpression of SOX2 gene by histone modifications: SOX2 enhances human prostate and breast Cancer progression by Prevention of apoptosis and enhancing cell proliferation. Oncology.

[15] Sanada Y, Yoshida K, Ohara M, Oeda M, Konishi K, Tsutani Y (2006). Histopathologic Evaluation of Stepwise Progression of Pancreatic Carcinoma with Immunohistochemical Analysis of Gastric Epithelial Transcription Factor SOX2: comparison of expression patterns between Invasive Components and cancerous or nonneoplastic int. Pancreas.

[16] Herreros-Villanueva M, Zhang J-S, Koenig A, Abel EV, Smyrk TC, Bamlet WR, de Narvajas AA-M, Gomez TS, Simeone DM, Bujanda L (2013). SOX2 promotes dedifferentiation and imparts stem cell-like features to pancreatic Cancer cells. Oncogenesis.

[17] Sanada Y, Yoshida K, Konishi K, Oeda M, Ohara M, Tsutani Y (2006). Expression of gastric mucin MUC5AC and gastric transcription factor SOX2 in Ampulla of Vater Adenocarcinoma: comparison between expression patterns and histologic subtypes. Oncol Rep.

[18] Wuebben EL, Wilder PJ, Cox JL, Grunkemeyer JA, Caffrey T, Hollingsworth MA, Rizzino A (2016). SOX2 functions as a Molecular Rheostat to control the growth, tumorigenicity and drug responses of pancreatic ductal adenocarcinoma cells. Oncotarget.

[19] Zhao H, Yan G, Zheng L, Zhou Y, Sheng H, Wu L, Zhang Q, Lei J, Zhang J, Xin R (2020). STIM1 is a metabolic checkpoint regulating the Invasion and Metastasis of Hepatocellular Carcinoma. Theranostics.

[20] Liu B, Yu H-H, Ye H-L, Luo Z-Y, Xiao F (2015). Effects of Stromal Interacting Molecule 1 gene silencing by short hairpin RNA on the Biological Behavior of Human gastric Cancer cells. Mol Med Rep.

[21] Wang J-Y, Sun J, Huang M-Y, Wang Y-S, Hou M-F, Sun Y, He H, Krishna N, Chiu S-J, Lin S (2015). STIM1 overexpression promotes colorectal Cancer progression, cell motility and COX-2 expression. Oncogene.

[22] Huang H-K, Lin Y-H, Chang H-A, Lai Y-S, Chen Y-C, Huang S-C, Chou C-Y, Chiu W-T (2020). Chemoresistant Ovarian Cancer enhances its Migration abilities by increasing Store-operated ca(2+) entry-mediated turnover of focal adhesions. J Biomed Sci.

[23] Liang X, Zhang N, Pan H, Xie J, Han W (2021). Development of store-operated calcium entry-targeted compounds in Cancer. Front Pharmacol.

[24] Alhamed AS, Alqinyah M, Alsufayan MA, Alhaydan IA, Alassmrry YA, Alnefaie HO, Algahtani MM, Alghaith AF, Alhamami HN, Albogami AM (2023). Blockade of store-operated calcium entry sensitizes breast Cancer cells to Cisplatin Therapy via modulating inflammatory response. Saudi Pharm J.

[25] Guo L, Mohanty A, Singhal S, Srivastava S, Nam A, Warden C, Ramisetty S, Yuan Y-C, Cho H, Wu X (2023). Targeting ITGB4/SOX2-Driven Lung Cancer Stem cells using proteasome inhibitors. iScience.

[26] Zhang S, Sun Y (2020). Targeting Oncogenic SOX2 in Human Cancer cells: therapeutic application. Protein Cell.

[27] Chun YS, Pawlik TM, Vauthey J-N (2018). 8th Edition of the AJCC Cancer staging Manual: pancreas and hepatobiliary cancers. Ann Surg Oncol.

[28] Nagtegaal ID, Odze RD, Klimstra D, Paradis V, Rugge M, Schirmacher P, Washington KM, Carneiro F, Cree IA (2020). The 2019 WHO classification of Tumours of the Digestive System. Histopathology.

[29] Hwang HK, Kim H-I, Kim SH, Choi J, Kang CM, Kim KS, Lee WJ (2016). Prognostic impact of the Tumor-Infiltrating Regulatory T-Cell (Foxp3(+))/Activated cytotoxic T lymphocyte (Granzyme B(+)) ratio on Resected Left-Sided Pancreatic Cancer. Oncol Lett.

[30] Chrysovergis A, Papanikolaou VS, Tsiambas E, Ragos V, Peschos D, Kyrodimos E (2019). Digital Analysis of BCL2 expression in laryngeal squamous cell carcinoma. Anticancer Res.

[31] Song S, Wang B, Gu S, Li X, Sun S (2017). Expression of Beclin 1 and Bcl-2 in pancreatic neoplasms and its effect on pancreatic ductal adenocarcinoma prognosis. Oncol Lett.

[32] Zarella MD, Heintzelman RC, Popnikolov NK, Garcia FU (2018). BCL-2 expression aids in the immunohistochemical prediction of the Oncotype DX breast Cancer recurrence score. BMC Clin Pathol.

[33] Kondratska K, Kondratskyi A, Yassine M, Lemonnier L, Lepage G, Morabito A, Skryma R, Prevarskaya N (2014). Orai1 and STIM1 mediate SOCE and contribute to apoptotic resistance of pancreatic adenocarcinoma. Biochim Biophys Acta.

[34] Tang J, Ye S, Wang M, Li J, Meng X, Liu F (2020). Stromal Interaction Molecule 1 promotes Tumor Growth in Esophageal squamous cell carcinoma. Genomics.

[35] Jia Y, Gu D, Wan J, Yu B, Zhang X, Chiorean EG, Wang Y, Xie J (2019). The role of GLI-SOX2 Signaling Axis for Gemcitabine Resistance in Pancreatic Cancer. Oncogene.

[36] Xie J, Pan H, Yao J, Zhou Y, Han WSOCE (2016). Cancer: recent progress and New perspectives. Int J cancer.

[37] Zhu D, He R, Yu W, Li C, Cheng H, Zhu B, Yan J (2021). ORAI3 contributes to Hypoxia-Inducible factor 1/2α-Sensitive Colon cell Migration. Physiol Int.

[38] Li Y, Guo B, Xie Q, Ye D, Zhang D, Zhu Y, Chen H, Zhu B (2015). STIM1 mediates Hypoxia-Driven Hepatocarcinogenesis via Interaction with HIF-1. Cell Rep.

[39] Chong Y, Thakur N, Paik KY, Lee EJ, Kang CS (2020). Prognostic significance of stem Cell/ Epithelial-Mesenchymal Transition Markers in Periampullary/Pancreatic cancers: FGFR1 is a promising prognostic marker. BMC Cancer.

[40] Novak D, Hüser L, Elton JJ, Umansky V, Altevogt P, Utikal J (2020). SOX2 in Development and Cancer Biology. Semin Cancer Biol.

[41] Wuebben EL, Rizzino A (2017). The Dark side of SOX2: Cancer - a comprehensive overview. Oncotarget.

[42] Nappo G, Funel N, Laurenti V, Stenner E, Carrara S, Bozzarelli S, Spaggiari P, Zerbi A (2023). Ampullary Cancer: histological subtypes, markers, and clinical behaviour-state of the art and perspectives. Curr Oncol.

[43] Ferchichi M, Jouini R, Koubaa W, Khanchel F, Helal I, Hadad D, Bibani N, Chadli-Debbiche A, BenBrahim E (2019). Ampullary and pancreatic Adenocarcinoma-a comparative study. J Gastrointest Oncol.

[44] Huang T-Y, Lin Y-H, Chang H-A, Yeh T-Y, Chang Y-H, Chen Y-F, Chen Y-C, Li C-C, Chiu W-T. STIM1 knockout enhances PDGF-Mediated ca(2+) signaling through Upregulation of the PDGFR^–^PLCγ^–^STIM2 Cascade. Int J Mol Sci. 2018;19(6). 10.3390/ijms19061799. 10.3390/ijms19061799 PMC6032054 29912163

[45] Wang Y, Wang H, Pan T, Li L, Li J, Yang H (2017). STIM1 silencing inhibits the Migration and Invasion of A549 cells. Mol Med Rep.

[46] Yang S, Huang X-Y (2005). Ca2 + influx through L-Type Ca2 + channels controls the Trailing tail contraction in growth factor-Induced Fibroblast Cell Migration. J Biol Chem.

[47] Kasashima H, Duran A, Martinez-Ordoñez A, Nakanishi Y, Kinoshita H, Linares JF, Reina-Campos M, Kudo Y, L’Hermitte A, Yashiro M (2021). Stromal SOX2 Upregulation promotes tumorigenesis through the generation of a SFRP1/2-Expressing Cancer-Associated Fibroblast Population. Dev Cell.

[48] Paterson C, Kilmister EJ, Brasch HD, Bockett N, Patel J, Paterson E, Purdie G, Galvin S, Davis PF, Itinteang T (2021). Cell populations expressing stemness-Associated markers in Lung Adenocarcinoma. Life (Basel Switzerland).

[49] Ram R, Brasch HD, Dunne JC, Davis PF, Tan ST, Itinteang T (2017). The identification of three Cancer stem cell subpopulations within moderately differentiated lip squamous cell carcinoma. Front Surg.

[50] Brar G, Blais EM, Joseph Bender R, Brody JR, Sohal D, Madhavan S, Picozzi VJ, Hendifar AE, Chung VM, Halverson D (2019). Multi-omic molecular comparison of primary versus metastatic pancreatic tumours. Br J Cancer.

[51] Zeng W, Yuan JP, Kim MS, Choi YJ, Huang GN, Worley PF, Muallem S (2008). STIM1 Gates TRPC channels, but not Orai1, by Electrostatic Interaction. Mol Cell.

[52] Xu J-M, Zhou Y, Gao L, Zhou S-X, Liu W-H, Li X-A (2016). Stromal Interaction Molecule 1 plays an important role in gastric Cancer progression. Oncol Rep.

[53] Hüser L, Novak D, Umansky V, Altevogt P, Utikal J (2018). Targeting SOX2 in Anticancer Therapy. Expert Opin Ther Targets.

[54] Bulle A, Lim K-H (2020). Beyond just a tight fortress: contribution of stroma to epithelial-mesenchymal transition in pancreatic Cancer. Signal Transduct Target Ther.

[55] Bhattacharya A, Kumar J, Hermanson K, Sun Y, Qureshi H, Perley D, Scheidegger A, Singh BB, Dhasarathy A (2018). The Calcium Channel Proteins ORAI3 and STIM1 mediate TGF-β Induced Snai1 expression. Oncotarget.

[56] Pan X, Cang X, Dan S, Li J, Cheng J, Kang B, Duan X, Shen B, Wang Y-J (2016). Site-specific disruption of the Oct4/Sox2 protein Interaction reveals coordinated Mesendodermal differentiation and the epithelial-mesenchymal transition. J Biol Chem.

[57] Belotte J, Fletcher NM, Alexis M, Morris RT, Munkarah AR, Diamond MP, Saed GM (2015). Sox2 gene amplification significantly impacts overall survival in Serous Epithelial Ovarian Cancer. Reprod Sci.

[58] Tang K, Liu J, Liu B, Meng C, Liao J (2022). SOX2 contributes to Invasion and poor prognosis of gastric Cancer: a Meta-analysis. Med (Baltim).

[59] Zhang S, Xiong X, Sun Y (2020). Functional characterization of SOX2 as an Anticancer Target. Signal Transduct Target Ther.

[60] Hosseini-Khah Z, Babaei MR, Tehrani M, Cucchiarini M, Madry H, Ajami A, Rakhshani N, Rafiei A, Nikbin B (2021). SOX2 and Bcl-2 as a Novel Prognostic Value in Hepatocellular Carcinoma Progression. Curr Oncol.

[61] Vervliet T, Parys JB, Bultynck G (2016). Bcl-2 proteins and Calcium Signaling: complexity beneath the Surface. Oncogene.

[62] Annovazzi L, Mellai M, Caldera V, Valente G, Schiffer D (2011). SOX2 expression and amplification in Gliomas and Glioma Cell lines. Cancer Genomics Proteom.

[63] Wilbertz T, Wagner P, Petersen K, Stiedl A-C, Scheble VJ, Maier S, Reischl M, Mikut R, Altorki NK, Moch H (2011). SOX2 gene amplification and Protein Overexpression Are Associated with Better Outcome in squamous cell Lung Cancer. Mod Pathol off J United States Can Acad Pathol Inc.

[64] Zhang Z, Liu X, Feng B, Liu N, Wu Q, Han Y, Nie Y, Wu K, Shi Y, Fan D (2015). STIM1, a direct target of MicroRNA-185, promotes Tumor Metastasis and is Associated with Poor Prognosis in Colorectal Cancer. Oncogene.

[65] Li Y, Du M, Wang S, Zha J, Lei P, Wang X, Wu D, Zhang J, Chen D, Huang D et al. Clinicopathological Implication of Long Non-Coding RNAs SOX2 Overlapping Transcript and Its Potential Target Gene Network in Various Cancers. *Frontiers in genetics*. Switzerland. 2019, p 1375. 10.3389/fgene.2019.01375. 10.3389/fgene.2019.01375 PMC6989546 32038720

[66] Zhu Y, Huang S, Chen S, Chen J, Wang Z, Wang Y, Zheng H (2021). SOX2 promotes Chemoresistance, Cancer Stem cells Properties, and epithelial-mesenchymal transition by β-Catenin and Beclin1/Autophagy signaling in Colorectal Cancer. Cell Death Dis.

