# Supplementary material for: STIM1/SOX2 proteins are co-expressed in the tumor and microenvironmental stromal cells of pancreatic ductal adenocarcinoma and ampullary carcinoma
Source: World J Surg Oncol. 2024 Mar 26;22:84. doi: 10.1186/s12957-024-03356-y (PMC10964627; doi:10.1186/s12957-024-03356-y)
Supplement: Supplementary file 1 — Supplementary Material 1: Table 1: Comparative between PDAC and AAC regarding the clinicopathological data [file 12957_2024_3356_MOESM1_ESM.docx]

**Supplementary table (1):** Comparative between PDAC and AAC regarding the **c**linicopathological data

| **Variables** | | **PDAC** | | **AAC** | | **Test of sig.** | **p-value** |
| --- | --- | --- | --- | --- | --- | --- | --- |
|  |  | **(No= 48)** | | **(No= 21)** | |  |  |
|  |  | **No.** | **%** | **No.** | **%** |  |  |
| **Age (years)** | <56 | 22 | 45.8 | 12 | 57.1 | χ^2^=0.748 | 0.387 |
|  | ≥56 | 26 | 54.2 | 9 | 42.9 |  |  |
| **Gender** | Male | 35 | 72.9 | 14 | 66.7 | χ^2^=0.277 | 0.599 |
|  | Female | 13 | 27.1 | 7 | 33.3 |  |  |
| **CA19-9 (U/mL)** | <200 | 16 | 33.3 | 4 | 33.3 | χ^2^=2.620 | 0.106 |
|  | ≥200 | 10 | 20.8 | 8 | 66.7 |  |  |
| **Tumor recurrence** | Present | 17 | 70.8 | 6 | 54.5 | χ^2^=0.888 | ^FE^p=0.451 |
|  | Absent | 7 | 29.2 | 5 | 45.5 |  |  |
| **Tumor size** | Mean ± SD. | 4.30±1.68 | | 2.59±1.17 | | U=192.50^*^ | <0.001^*^ |
|  | Median | 4 | | 3 | |  |  |
| **Histologic grade** | GI | 10 | 20.8 | 3 | 14.3 | χ^2^=0.936 | ^MC^p=0.630 |
|  | GII | 33 | 68.8 | 17 | 81 |  |  |
|  | GIII | 5 | 10.4 | 1 | 4.8 |  |  |
| **LVI** | Present | 15 | 31.3 | 5 | 23.8 | χ^2^=0.393 | 0.531 |
|  | Absent | 33 | 68.8 | 16 | 76.2 |  |  |
| **Perineural invasion** | Present | 47 | 97.9 | 10 | 47.6 | χ^2^=25.725^*^ | ^FE^p<0.001^*^ |
|  | Absent | 1 | 2.1 | 11 | 52.4 |  |  |
| **Resection margins** | involved | 23 | 47.9 | 2 | 9.5 | χ^2^=9.320^*^ | 0.002^*^ |
|  | Free | 25 | 52.1 | 19 | 90.5 |  |  |
| **Pathological stage** | Early | 29 | 60.4 | 10 | 47.6 | χ^2^=1.974 | ^MC^p=0.324 |
|  | Late | 19 | 39.6 | 11 | 52.4 |  |  |
| **LN**s **status** | Positive | 35 | 72.9 | 11 | 52.4 | χ^2^=2.772 | 0.096 |
|  | Negative | 13 | 27.1 | 10 | 47.6 |  |  |
| **Epithelial desmoplasia ratio** | Epithelial | 19 | 39.6 | 19 | 90.5 | χ^2^=15.423^*^ | <0.001^*^ |
|  | Desmoplasia | 21 | 43.8 | 1 | 4.8 |  |  |
|  | Equal | 8 | 16.7 | 1 | 4.8 |  |  |
| **TIMCs** | Mean ± SD. | 9.75 ± 10.50 | | 8.62 ± 7.31 | | U=487.0 | 0.819 |
|  | Median | 5 | | 5 | |  |  |

**PDAC:** Pancreatic ductal adenocarcinoma **AAC:** Ampullary adenocarcinoma **SD:** Standard deviation**, CA19-9**: Carbohydrate antigen 19-9**, LVI:** Lymphovascular invasion, **LNs:** Lymph nodes**, T:** Tumor**, TIMCs:** Tumor infiltrating mononuclear cells, **χ^2^**: Chi square test, **FE:** Fisher Exact, **MC:** Monte Carlo,  **U**: Mann Whitney test, t: Student t-test.

p: p value for comparing between the two categories

*: Statistically significant at p ≤ 0.05
